# Anisotropic Crb accumulation, modulated by Src42A, is coupled to polarised epithelial tube growth in *Drosophila*

**DOI:** 10.1371/journal.pgen.1007824

**Published:** 2018-11-26

**Authors:** Ivette Olivares-Castiñeira, Marta Llimargas

**Affiliations:** Institut de Biologia Molecular de Barcelona, CSIC, Parc Científic de Barcelona, Baldiri Reixac, Barcelona, Spain; Harvard Medical School, Howard Hughes Medical Institute, UNITED STATES

## Abstract

The control of the size of internal tubular organs, such as the lungs or vascular system, is critical for proper physiological activity and to prevent disease or malformations. This control incorporates the intrinsic physical anisotropy of tubes to generate proportionate organs that match their function. The exact mechanisms underlying tube size control and how tubular anisotropy is translated at the cellular level are still not fully understood. Here we investigate these mechanisms using the *Drosophila* tracheal system. We show that the apical polarity protein Crumbs transiently accumulates anisotropically at longitudinal cell junctions during tube elongation. We provide evidence indicating that the accumulation of Crumbs in specific apical domains correlates with apical surface expansion, suggesting a link between the anisotropic accumulation of Crumbs at the cellular level and membrane expansion. We find that Src42A is required for the anisotropic accumulation of Crumbs, thereby identifying the first polarised cell behaviour downstream of Src42A. Our results indicate that Src42A regulates a mechanism that increases the fraction of Crb protein at longitudinal junctions, and genetic interaction experiments are consistent with Crb acting downstream of Src42A in controlling tube size. Collectively, our results suggest a model in which Src42A would sense the inherent anisotropic mechanical tension of the tube and translate it into a polarised Crumbs accumulation, which may promote a bias towards longitudinal membrane expansion, orienting cell elongation and, as a consequence, longitudinal growth at the tissue level. This work provides new insights into the key question of how organ growth is controlled and polarised and unveils the function of two conserved proteins, Crumbs and Src42A, with important roles in development and homeostasis as well as in disease, in this biological process.

## Author summary

The regulation of the size of tubular organs is critical to ensure their physiological activity and to prevent disease and malformations. Here we use the *Drosophila* tracheal system as a suitable model to investigate the mechanisms underlying size regulation of tubular organs. Different mechanisms were known to control the growth of tracheal tubes along the longitudinal axis, namely apical expansion mediated by Crumbs, polarised cell shape changes mediated by Src42A, and a restricting activity mediated by an apical extracellular matrix. However, it was unclear how the different mechanisms interacted and how they polarised tube growth. In this work we find that Crumbs accumulates in a polarised manner during tube elongation and that Src42A contributes to this anisotropic accumulation, identifying a polarised cellular behaviour downstream of Src42A. In addition, our results indicate a correlation between apical membrane expansion and Crb localisation in the SubApical Region, suggesting that this localisation could facilitate or promote apical membrane expansion. We propose a model where, in response to the intrinsic polarised tension of tubes, Src42A increases Crumbs accumulation in longitudinal cell junctions, which could mediate a polarised membrane expansion that would orient tube elongation.

## Introduction

Tubes are physically anisotropic, with a curved circumferential axis and a flat longitudinal one. This physical property confers orientation and polarisation to tubes, critical for their physiological activity in biological systems. Many vital organs, such as the lungs, vascular system or mammary glands, are internal tubular structures [1–3], underscoring the importance of investigating how they form and polarise to be functional.

The tracheal (respiratory) system of *Drosophila* is a paradigm for the analysis of tubular organs [4]. After a morphogenetic phase by branching morphogenesis [5,6] the tracheal tubes mature and become physiologically active [7]. Tube maturation ensures the acquisition of the correct tube diameter and length, and of gas filling, critical steps for organ functional activity [3,8,9]. The diametrical and longitudinal growth of tracheal tubes are two different events regulated by different mechanisms (reviewed in [10]). Tube longitudinal growth starts at the end of stage 14 and continues until the embryo hatches. Several mechanisms have been shown to control this elongation. These include the proper modification of the apical extracellular matrix, aECM, consisting of a transient filament made of chitin and chitin-associated proteins [11,12]), particularly Serpentine (Serp) and Vermiform (Verm), whose absence leads to tube overelongation [13,14]. Cell intrinsic mechanisms such as Crumbs (Crb)-mediated apical membrane growth [15,16] and Src42A-mediated cell shape regulation [17,18] also control tube length. A model of tube length control has been proposed [10,15,19] where the apical membrane expansion force driven by Crb is balanced with the aECM resistance through factors that attach the aECM to the apical membrane. Excess or defects in any of these two forces or their uncoupling leads to tube length defects. However, several questions remain unclear, such as how the Crb-mediated apical membrane growth is biased to the longitudinal direction, how the different factors interact, or how non polarised Src42A accumulation controls polarised cell shape changes.

## Results and discussion

### Crb accumulates anisotropically during tube elongation

**Fig 1:**
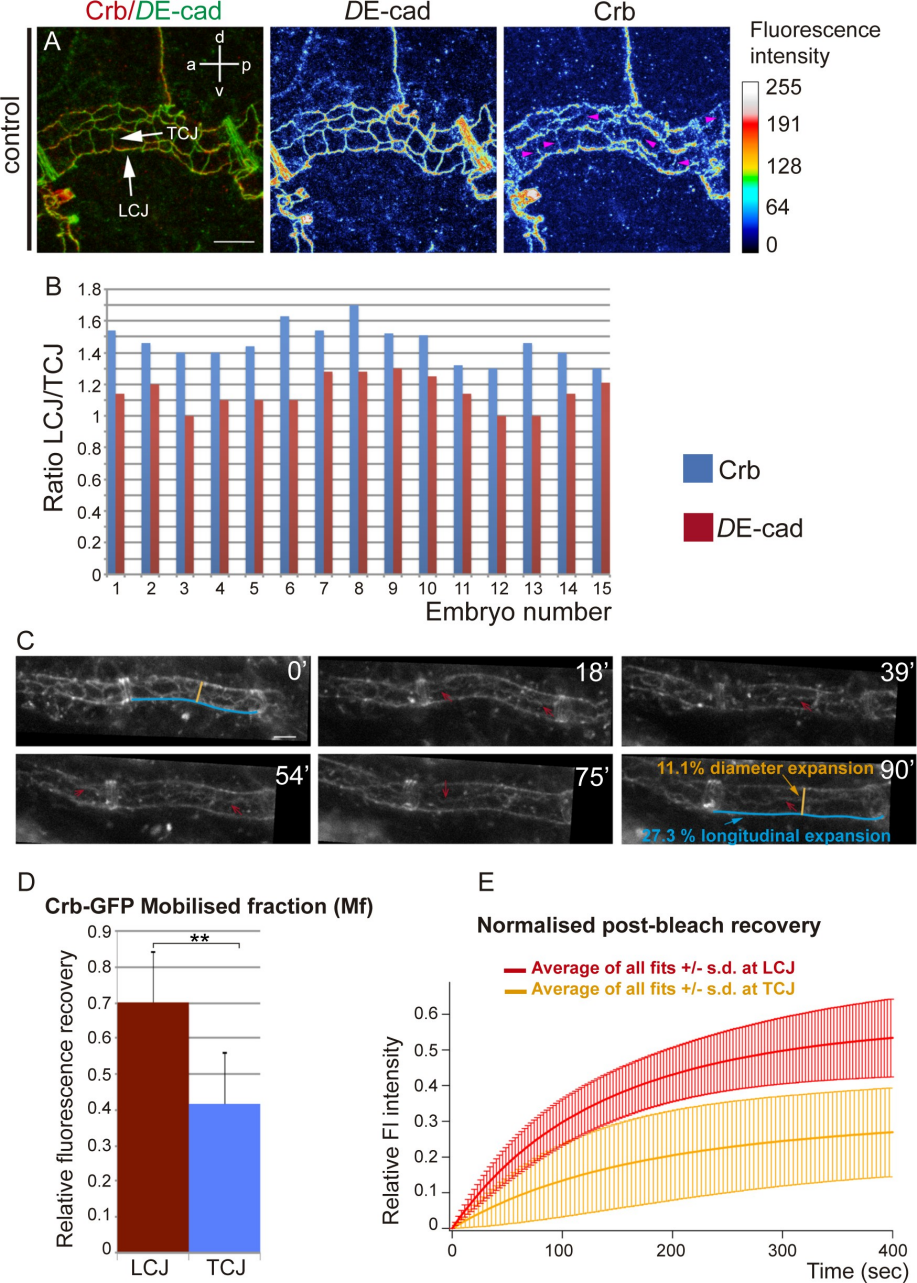
Anisotropic accumulation of Crb during tube elongation. (A) Confocal projection of a metamere of the DT of a stage 16 embryo stained for *D*E-cad and Crb. The fluorescence intensity of *D*E-cad and Crb is shown in heat maps. The fluorescence intensity values mapped to the gradient bar to the right ranging from black (0) to white (255). The gradient bar was generated with the “calibration bar” tool in ImageJ. Note that while *D*E-cad shows similar fluorescence intensity at longitudinal and transverse cell junctions (LCJs and TCJs respectively, white arrows), Crb fluorescence intensity is higher at LCJs. Crb accumulation at many TCJs is hardly detectable (pink arrowheads). Scale bar 7,5 μm (B) Quantification of the ratio of *D*E-cad and Crb accumulation at LCJs and TCJs for each embryo analysed. Note that while *D*E-cad ratio is close to 1, indicating an equal fluorescence intensity at all cell junctions, Crb ratio indicates increased fluorescence intensity in LCJs. n = 254 LCJs and n = 314 TCJs from 15 wild type embryos. (C) Stills from a time-lapse movie of the accumulation of Crb in the DT (using *Crb^GFP^*, a viable knock-in allele that provides the only source of Crb protein in the embryo) in an otherwise wild type background. Note the enrichments of Crb in LCJs (red arrows) at different time points (in minutes) during stage 16. Time point 0' corresponds to an embryo at the end of stage 15. Note also the increase in tube length during 1:30 h and the moderate increase in diameter. Scale bar 5 μm (D) The Mobile fraction, Mf, was calculated and compared for LCJs and TCJs experiments. Mf was significantly higher at LCJs than at TCJs. Error bars indicate standard error (s.e.).(E) Comparison of the average fit from the different FRAP experiments of normalised post-bleach recovery in LCJs and TCJs. Note the higher recovery at LCJs. Error bars indicate standard deviation (s.d.). n = 8 LCJs and n = 6 TCJs from *Crb*^*GFP*^ embryos.

Since Crb has been proposed to regulate tube length by promoting apical membrane growth [15,16], we first examined Crb accumulation in the Dorsal Trunk (DT, the main tracheal trunk connecting to the exterior through the spiracles). Crb can localise to different subdomains of the apical membrane during tracheal development: the SubApical Region (SAR) and the Apical Free Region (AFR) [20]. The AFR corresponds to the most apical domain, free of contact with other epithelial cells and in direct contact with the lumen in the case of tubular organs like the trachea, while the SAR corresponds to the most apicolateral membrane domain of contact between neighboring epithelial cells. We previously showed that during the stages of higher longitudinal DT growth, stage 15 onwards, Crb accumulated strongly in the SAR, displaying a mesh-like pattern that identifies the apical junctional domain [20]. Strikingly, we now observed that Crb was anisotropically (not uniformly) distributed in the SAR of cell junctions. We classified cell junctions as longitudinal cell junctions (LCJs), mainly parallel to the longitudinal axis of the tube, and transverse cell junctions (TCJs), perpendicular to the longitudinal axis (see Materials and Methods). We found that Crb accumulation was more visible at LCJs than at TCJs (Fig 1A, S1A Fig), observing several examples where accumulation of Crb at TCJs was almost absent (Fig 1A pink arrowheads). We quantified the accumulation of Crb (total fluorescence intensity/junctional length) at LCJs and TCJs and found that Crb accumulation was biased to LCJs, where levels were around 30% higher than at TCJs (average % of difference of Crb accumulation at LCJs and TCJs, n = 15 embryos). To compare different embryos we calculated the LCJ/TCJ ratio of Crb accumulation (Fig 1B), which showed an average of 1,5 (n = 15 embryos), indicating that Crb is anisotropically distributed, i.e. polarised. In contrast to Crb, *D*E-Cadherin (*D*E-cad), a core component of the Adherens Junctions (AJs), was equally distributed among all cell junctions (Fig 1A, S1A Fig). The ratio of accumulation in LCJ/TCJ was close to 1 (Fig 1B), indicating that the anisotropic distribution is not a general feature of all junctional proteins. These results indicated that a larger proportion of LCJs accumulate higher levels of Crb than TCJs.

To further investigate this observation we carried out time-lapse imaging in embryos carrying the viable and functional *Crb*^*GFP*^ allele as the only source of functional Crb protein (S1 Movie). We observed enrichments of Crb protein at LCJs, and less conspicuous accumulations at TCJs, from late stage 15 and during stage 16 over a period of 1,30–2 hours (red arrows in Fig 1C). This correlated with an increase in tube length of around a 30% and a moderate increase in tube diameter of an 11% (n = 4 movies).

Altogether these results point to a polarised accumulation of Crb that correlates with an anisotropic growth along the longitudinal axis of the DT during stage 16. It is worth pointing out that anisotropies of Crb, like the one described here, or of other apical determinants, have important implications in morphogenesis [21,22].

### Differential turnover of Crb protein at cell junctions

Different molecular mechanisms could underlie the preferential accumulation of Crb at LCJs, such as specific Crb degradation at TCJs, specific stabilisation at LCJs, targeted intracellular trafficking, differential protein recycling, among others. To investigate the possible mechanism behind the anisotropic pattern of Crb accumulation we performed FRAP analysis at either LCJs or TCJs of embryos carrying the *Crb*^*GFP*^ allele (S2D–S2G Fig, S2 and S3 Movies). We found that the amount of fluorescent protein, relative to the pre-bleach value, mobilized during the experimental time (mobile fraction, Mf) was significantly higher at LCJs compared to TCJs, indicating a higher recovery of CrbGFP protein at LCJs (Fig 1D and 1E, S2A and S2C Fig). To assess the recovery kinetics we calculated the half-time (t_1/2_, time to reach half of the Mf). We found that the half-time was not significantly different at LCJs and TCJs, suggesting that the recovery rate is comparable at the differently oriented junctions (S2B Fig). Kymographs of the bleached regions suggested that the recovery was not due to lateral diffusion (S2E and S2G Fig). Altogether our results indicated a higher mobility of Crb protein at LCJs but a constant rate of incorporation in all junctions.

Hence, on the one hand we find that higher levels of Crb accumulate at LCJs, and on the other, FRAP experiments show that Crb protein is more mobile at LCJs. These results could suggest the existence of two molecularly defined different pools of Crb in the junctions with different mobility: a basal level-pool with lower mobility and an enrichment-pool with higher mobility. The basal level-pool would be present in all junctions, while a mechanism acting specifically at LCJs would ensure also the presence of the enrichment-pool there. The increased mobility/instability of the enrichment-pool of Crb at LCJs would contribute to increase the total Crb mobility at LCJs. Further experiments will be required to test this possibility and to understand how the molecular mechanism underlying the increased accumulation of Crb at LCJs relates to the differential mobility of Crb protein that we document.

### Src42A is required for anisotropic Crb accumulation

**Fig 2:**
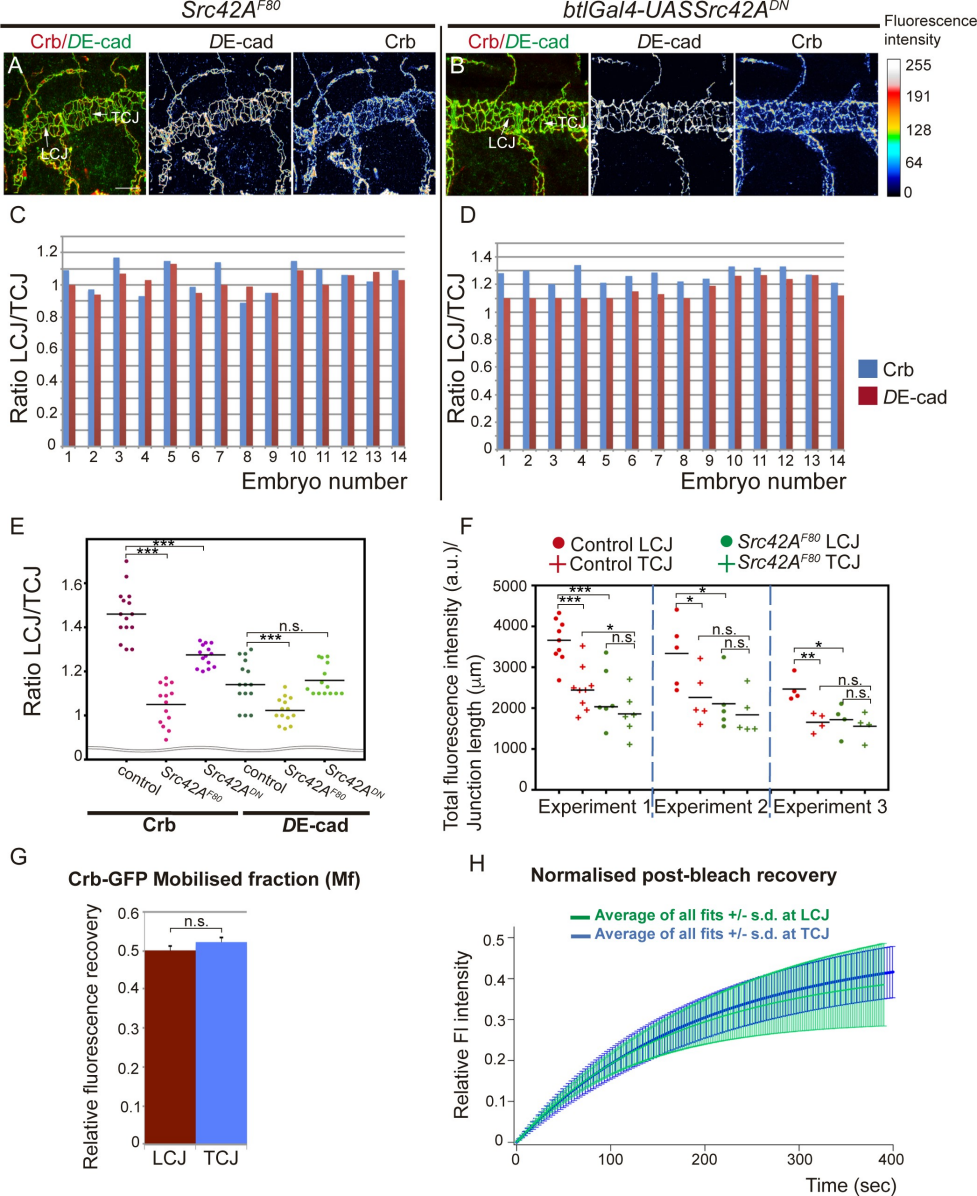
Src42A modulates Crb anisotropic accumulation. (A,B) Confocal projections of the DT of stage 16 *Src42A* mutants (A) or in embryos in which Src42A is downregulated in the tracheal cells (B). Embryos are stained for *D*E-cad and Crb. The colour-coded fluorescence intensity shown for *D*E-cad and Crb matches the heat map shown on the right. Note the homogeneity of fluorescence intensities at LCJs and TCJs for *D*E-cad and also for Crb. Scale bar 10 μm (C,D) Quantification of the ratio of *D*E-cad and Crb accumulation at LCJs and TCJs in each embryo analysed in *Src42A* mutants (C) or in Src42A is downregulation conditions (D). n = 288 LCJs and n = 297 TCJs from 14 *Src42A*^*F80*^ mutant embryos, n = 349 LCJs and n = 417 TCJs from 14 *Src42A*^*DN*^embryos. (E) Scatter Plot comparing the LCJ/TCJ ratio of accumulation of Crb and *D*E-cad in control, *Src42A*^*F80*^ and *Src42A*^*DN*^embryos. Note that while the ratio of *D*E-cad is close to 1 in control and mutants, the ratio of LCJ/TCJ Crb accumulation is significantly different in the control and mutant conditions. (F) Scatter Plot comparing the total levels of Crb protein at LCJs and TCJs (normalised to junction length) in control and *Src42A*^*F80*^ mutant embryos. Measurements were performed in three different independent experiments in which mutant and control embryos were processed together. Note that in all experiments the levels of Crb at LCJs in *Src42A*^*F80*^*/Cyo* heterozygotes (control embryos) are higher than those of TCJs and than those at LCJs in the *Src42A*^*F80*^ mutant sibling embryos. The levels in LCJs and TCJs of mutants are similar to those of TCJs of control embryos. (G) The Mobile fraction in LCJs and TCJs was comparable. Error bars indicate standard error (s.e.). (H) Comparison of the average fit of normalised post-bleach recovery from different experiments in LCJs and TCJs. Note the similar recovery in LCJs and TCJs. Error bars indicate standard deviation (s.d.).n = 7 LCJs and n = 9 TCJs from mutant embryos.

We next asked how the anisotropic distribution of Crb is regulated. To investigate this question we turned our attention to Src42A, as it triggers one of the mechanisms regulating tube elongation, orienting membrane growth on the longitudinal axis. In conditions of Src42A loss of function, LCJs do not expand and tubes become shorter [17,18]. We analysed Crb accumulation in loss of function conditions for Src42A. On the one hand we used the *Src42A*^*F80*^ allele, which lacks the distinct accumulation of phosphorylated Src42A (pSrc42A) at the apical junctional region but does not affect the stability or membrane localisation of the protein (S1F and S1G Fig and [17]). This mutation renders a kinase non-activatable protein that was previously shown to strongly affect tracheal tube elongation [17]. On the other hand we expressed a kinase-dead dominant negative form of Src42A (Src42^DN^) in the trachea, also previously shown to affect tube elongation [17,18]. In both cases we observed a more uniform distribution of Crb at LCJs and TCJs (Fig 2A and 2B, S1B and S1C Fig). Quantification of Crb levels indicated that the differences between the accumulation of Crb at LCJs compared to TCJs were reduced. Analysis of the LCJ/TCJ ratio of Crb clearly showed a significant decrease when compared to the control (Fig 2C–2E), indicating a more uniform accumulation in Src42A loss of function conditions. *D*E-cad LCJ/TCJ ratio in Src42A loss of function conditions remained close to 1, indicating a homogeneous distribution (Fig 2C–2E). Altogether these results show that a decrease in Src42A activity leads to a decrease of the anisotropic accumulation of Crb.

To investigate whether Src42A promotes an increased accumulation of Crb protein at LCJs or a depletion at TCJs we quantified the total levels of protein accumulation at LCJs and TCJs and compared control (i.e. heterozygotes) and *Src42A* mutants (i.e. *Src42A*^*F80*^ homozygotes) from the same experiment. While we observed variability within each genotypic group, different independent experiments indicated that in control embryos there is an increased accumulation of Crb protein at LCJs that is lost in *Src42A*^*F80*^ mutants (Fig 2F). In *Src42A* mutant conditions the levels of Crb accumulation at LCJs and TCJs were similar to those of TCJs of control embryos, indicating that Src42A regulates a mechanism that increases the fraction of Crb protein at LCJs.

Consistent with a role for Src42A in regulating directly or indirectly Crb accumulation we found partial co-localisation of Crb and Src42A protein, and with pSrc42A at the SAR (S1D and S1E Fig). However, we could not detect polarised accumulation of the active pSrc42A fraction during tube elongation (S1H Fig), as previously documented [17]. While we cannot discard transient anisotropies of pSrc42A accumulation that we cannot detect with the available antibodies, this result suggests that other factors (e.g. mechanical or chemical) modulate the activity of pSrc42A in the different junctions to regulate the anisotropic accumulation of Crb.

To further explore Src42A requirement we performed FRAP experiments in *Crb*^*GFP*^ embryos in which Src42A was downregulated (S2H–S2K Fig, S4 and S5 Movies). We found clear differences with respect to control: while in the control the Mf and recovery curves of LCJs and TCJs were clearly different (Fig 1E–1H, S2A and S2C Fig), in *Src42*^*DN*^ conditions the Mf and recovery curves of LCJs and TCJs were comparable (Fig 2G and 2H, S2A, S2C and S2H–S2K Fig). The Mf at the LCJs of Src42^DN^ was significantly lower than the Mf at the LCJs in control embryos, and was similar to the Mf at TCJs in control and mutant embryos (S2A Fig). The halftime recovery, t_1/2_, was comparable to that of control embryos, indicating a recovery rate similar in all cases (S2B Fig).

Altogether our results indicate that Src42A contributes to Crb preferential enrichment at LCJs and that it increases Crb mobility there. The fact that Crb levels and Crb recovery are affected particularly at LCJs when Src42A is downregulated strongly suggests that Src42A is (more) active precisely at LCJs, as previously suggested [17,18]. Our results are consistent with the proposed model (see above) in which a mechanism acting specifically at LCJs, that we now propose is mediated by Src42A, would ensure the accumulation of an enrichment-pool of Crb at LCJs with high protein mobility. In the absence of this Src42A-mediated activity, only the basal level-pool of Crb would be present at LCJs and TCJs, leading to comparable levels and mobility of Crb in all junctions. Src42A would not regulate the basal level-pool of Crb, and would instead be required to top up Crb at LCJs with an enrichment-pool of Crb. Future experiments addressing the molecular mechanism by which Src42A regulates Crb accumulation and its mobility will help to fully understand how it regulates the anisotropic accumulation of Crb at LCJs. Src42A-independent accumulation of Crb in tracheal cells together with other Src42A-independent mechanisms of apical membrane growth may be responsible for tube growth in the absence of Src42A.

### Regulation of tube elongation by activation of Src42A

**Fig 3:**
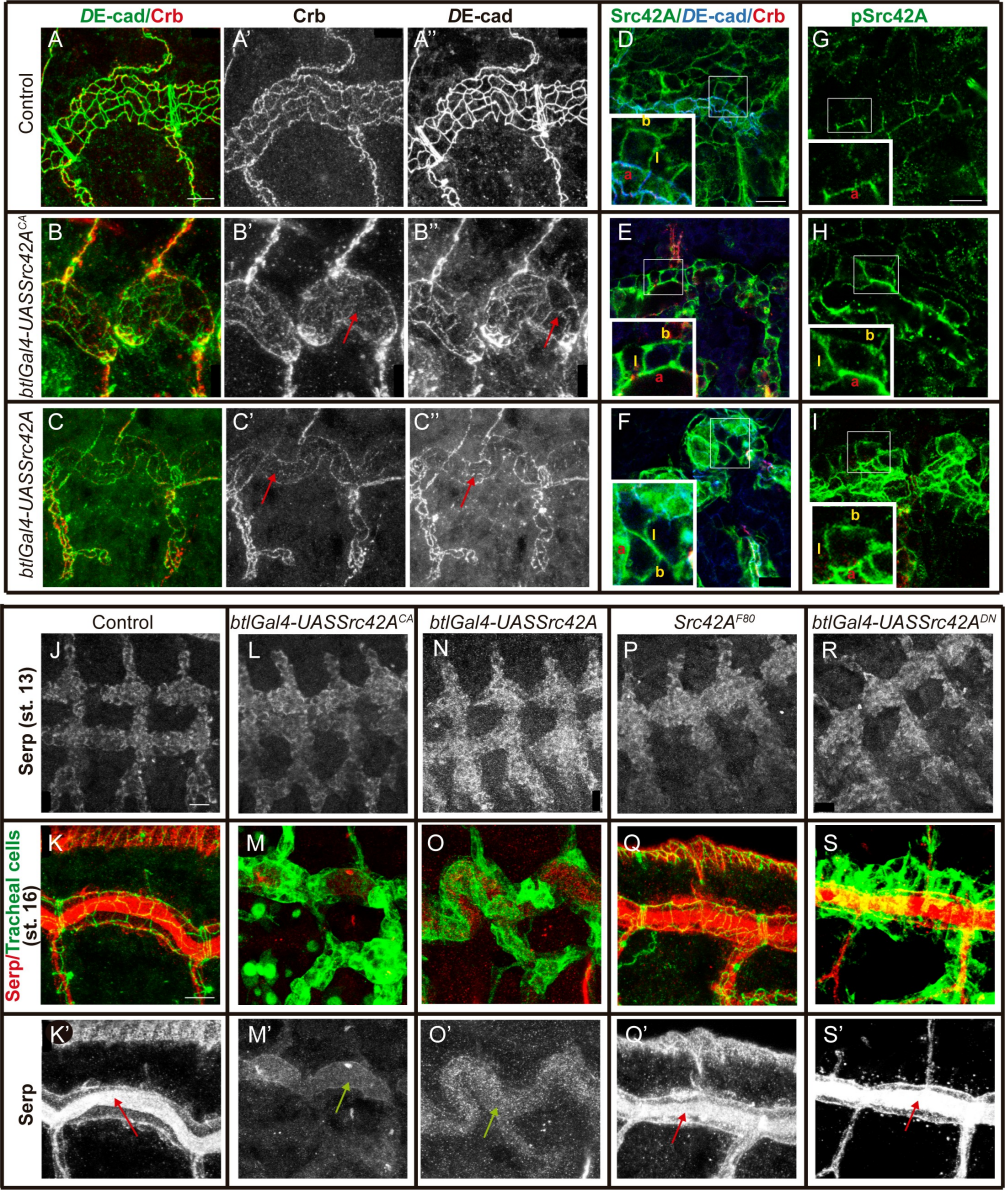
Src42A overactivation affects the accumulation of proteins that undergo endocytic trafficking in the tracheal system. Lateral views showing a region of the DT of embryos of indicated genotypes stained for the indicated markers. (A-C) Note that Crb accumulation is decreased in the DT (red arrows in B' and C') and *D*E-cad staining is fragmented and decreased in AJs (red arrows in B'' and C'') in embryos expressing constitutively active Src42A (B) or in Src42A overexpression conditions (C). Scale bar 7,5 μm (D-F) Src42A protein accumulates to cell membranes in control (D) and Src42A overactivation (E) or overexpression conditions (F). Insets (corresponding to close-ups of the regions marked by rectangles) show the accumulation in the apical (a), lateral (l) and basal (b) domains of the membrane. Images show single confocal sections. Scale bar 10 μm (G-I) In contrast to Src42A protein, activated pSrc42A protein accumulates exclusively to the apical region (a) of the membrane in the control (G), but expands along the lateral (l) and basal (b) membrane in Src42A overactivation (H) or overexpression conditions (I). Insets (corresponding to close-ups of the regions marked by rectangles) show the accumulation in the membrane domains. Images show single confocal sections. Scale bar 7,5 μm (J-S) Images show accumulation of Serp (red or white) in tracheal cells (marked in green with DE-cad, K,Q, or GFP in embryos carrying *btlGal4-UAS-Src-GFP*, M,O,S). Serp is accumulated normally in tracheal cells at early stages in all conditions analysed (J,L,N,P,R). At st 16 Serp is normally accumulated in the luminal compartment associated with the chitin filament (red arrows) in control (K,K') and *Src42A* loss of function conditions (Q,Q',S,S'). This accumulation is lost (green arrows) in Src42A overactivation (M,M') or overexpression conditions (O,O'). Scale bar J 10 μm, K 7,5 μm.

We found that Src42A is required for the anisotropic accumulation of Crb at LCJs. We then asked whether the overelongation of tubes observed in Src42A overactivation conditions (either overexpression of a wild type form of Src42A or expression of a constitutively active protein) [17,18] was due to an increased accumulation of Crb at LCJs. Our results did not support this expectation. We found that Crb was strongly decreased in the SAR of DT cells both in conditions of overexpression (*UASSrc42A*) or constitutive activation (*UASSrc42A*^*CA*^) (Fig 3A–3C). In conditions of mild overexpression of wild type Src42A, we found rare cases (around 5–8% of embryos) where we could detect some levels of Crb in the SAR, which accumulated preferentially at LCJs, as expected (S3A Fig). These results suggested that the tube length defects produced by Src42A overexpression/overactivation were caused by a mechanism different than the one operating in physiological conditions. To investigate this possible mechanism we analysed the levels and distribution of the total Src42A protein and the pSrc42A active fraction in both Src42A overexpression and overactivation conditions. We found that levels of Src42A protein were increased but still enriched in the membrane region (Fig 3D–3F). Interestingly, pSrc42A was not restricted anymore to the junctional apical region as in the wild type (Fig 3G) and instead it was expanded along the whole apicobasal membrane (Fig 3H and 3I). The increase and expansion of pSrc42A accumulation observed in overexpression and in overactivation conditions indicate that Src42A activity is overactivated in both cases and may explain the similarity of phenotypes. Further analysis also indicated that Src42A overexpression/overactivation leads to a general loss of cell organisation and membrane polarity, as evidenced by the miss-localisation of markers of membrane polarity, like the Septate Junction protein Megatrachea [23] (S3B and S3C Fig). These results indicate that an unregulated accumulation of active pSrc42A leads to a generalised miss-organisation of the cell and prevents proper Crb accumulation.

To investigate the cause of tube overelongation found in Src42A overexpression /overactivation conditions we analysed other known tube length regulators. One of them is Serp, which regulates the aECM organisation [13,14]. We found that in Src42A and Src42A^CA^ overexpression conditions Serp is lost from the luminal compartment (Fig 3K, 3M and 3O), although, as in wild type, tracheal cells accumulate Serp at early stages (Fig 3J, 3L and 3N). This result provides explanation for the tube elongation defects observed under these conditions, as Serp absence leads to tube overelongation. Interestingly, we could not detect defects in Serp accumulation in Src42 mutants or in Src42A^DN^ conditions (Fig 3P–3S), as previously reported [18]. These results suggested again that Src42A overactivation use a different mechanism than the one used in physiological conditions to drive tube elongation. Hence, our analysis of Src42A overactivation provides new results that allow to revisit and reinterpret previously published work [17,18].

Altogether our results indicate that an unregulated accumulation of active pSrc42A leads to a generalised miss-organisation of the cell and prevents proper accumulation of Crb and Serp (Fig 3B', 3C', 3M' and 3O'). In addition, we and others also observed that *D*E-cad was not properly localised either (Fig 3B'' and 3C'' and [17]). Interestingly, it has been shown that the tracheal accumulation of these proteins depends on their recycling [20,24,25]. Thus, our results could suggest a role of Src42A in protein trafficking. In this context, the loss of cell organisation and membrane polarity produced by mislocalisation of pSrc42A could interfere with protein trafficking. Roles for Src42A in protein trafficking have been proposed in different contexts [17,26–28]. Src42A could regulate protein trafficking directly, or indirectly through the regulation of the actin cytoskeleton. The actin cytoskeleton plays a capital role in protein trafficking [29] and Src42A acts as a regulator of the actin cytoskeleton [30,31]. A disruption of actin organisation in Src42A overactivation could lead to defects in the sorting of different cargoes as well as defects in endosomal maturation. Further experiments will be required to investigate a possible involvement of Src42A in protein trafficking during tracheal development.

### Crb accumulation in the SAR correlates with apical cell expansion

After identifying an anisotropic accumulation of Crb regulated by Src42A, we asked how this mechanism relates to tube elongation. Crb has been proposed to promote apical membrane growth independently of its role in apicobasal polarity at late stages of epithelial differentiation [32,33]. In the trachea Crb was proposed to mediate tube elongation by promoting apical membrane growth [16]. Interestingly, our results show an enrichment of Crb in the SAR of LCJ during tube elongation. This observation raises the hypothesis that it is precisely this accumulation of Crb in the SAR of LCJs what favours or facilitates apical membrane expansion (either by membrane growth or membrane transformation leading to cell shape changes), orienting cell elongation and as a consequence the longitudinal growth of the tube. Crb recycling during tracheal development could favour the mobilisation of cellular and/or membrane components facilitating membrane growth or membrane transformation. To investigate this possibility we analysed Crb accumulation in the SAR in different experimental conditions in which apicobasal polarity was unaffected.

**Fig 4:**
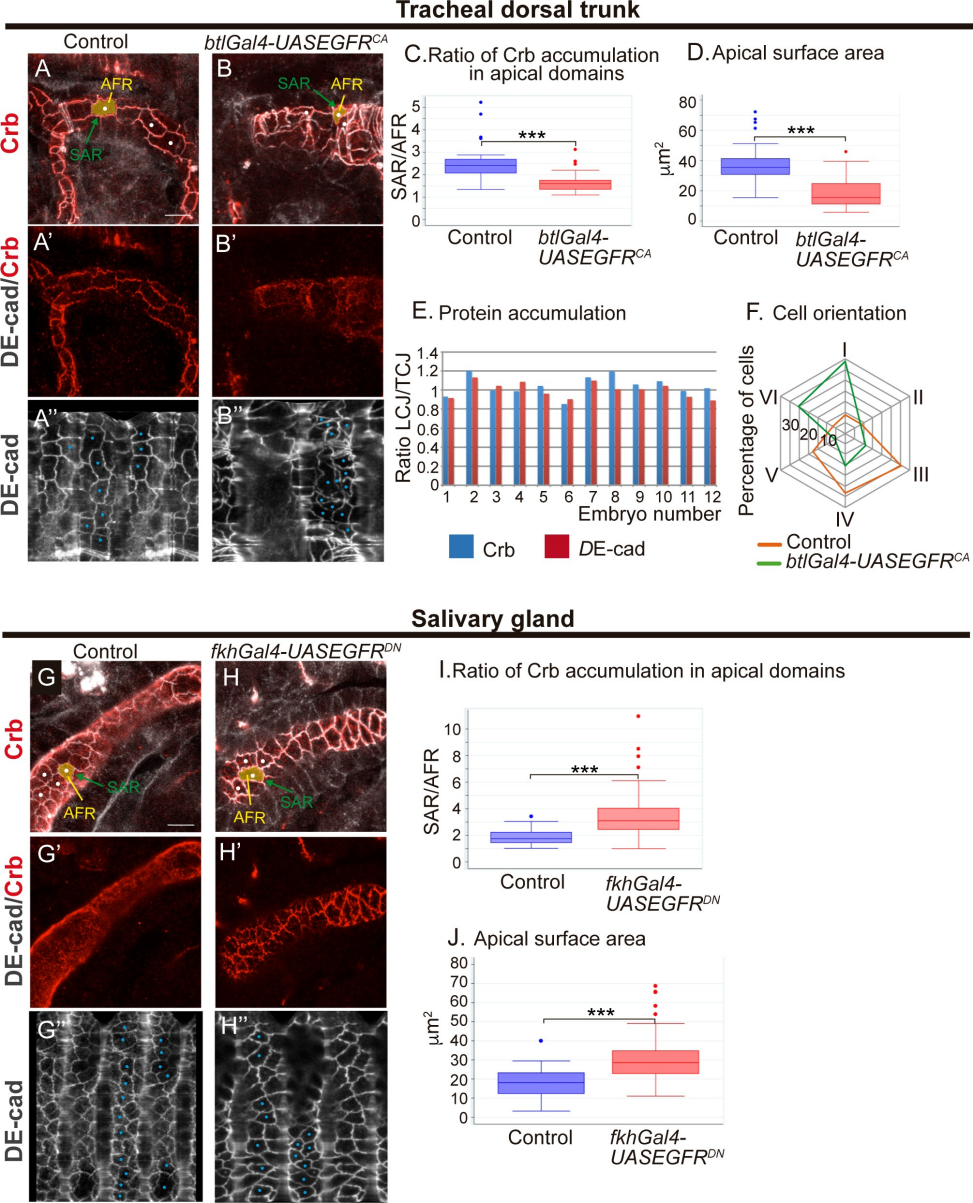
The subcellular localisation of Crb in apical domains correlate with apical expansion. (A,B) Lateral views showing a region of the DT of stage 16 embryos of indicated genotypes stained for the indicated markers. Images correspond to a single confocal section or to a projection of 2–3 sections to include all the apical domain of selected cells (marked with a white dot). The SAR outlines the cell contours and the AFR fills the cell space inside the SAR. (A',B') Note the poor enrichment of Crb in the SAR when EGFR is constitutively activated. (A'',B'') Tubes were unfolded and *D*E-cad staining was used to follow the junctional domain in order to measure the apical surface area in selected cells (blue dots). Scale bar 7,5 μm (C) Quantification of the ratio of Crb accumulation in the different apical regions (as published in [20]). n = 25 cells from 8 control embryos, n = 53 cells from 7 EGFR^CA^ embryos (D) Quantification of the cell area of the apical region. n = 46 cells from 5 control embryos, n = 71 cells from 5 EGFR^CA^ embryos (E) Quantification of the ratio of *D*E-cad and Crb accumulation at LCJs and TCJs for each embryo analysed. Note that Crb and *D*E-cad ratio is close to 1, indicating a similar intensity at all cell junctions. n = 163 LCJs and n = 160 TCJs from 12 EGFR^CA^ embryos. (F) Represents the orientation of DT cells. The radial chart indicates the proportion of cells included in intervals I-VI. In interval I cells are oriented from 0 to 30 degrees; in interval II from 30°-60°; in interval III from 60°-90°; in interval IV from 90°-120°; in interval V from 120°-150° and in interval VI from 150°-180°. Note the different orientation of DT cells in control and EGFR^CA^ conditions (G,H) Lateral views showing a region of the salivary gland of stage 16 embryos of indicated genotypes stained for the indicated markers. Images correspond to a single confocal section or to a projection of 2–3 sections to include all the apical domain of selected cells (marked with a white dot). Note that in control embryos Crb is not particularly enriched in the SAR (G'). In contrast, when EGFR is downregulated (H') Crb clearly relocalises and accumulates in the SAR. (G'',H'') Salivary glands were unfolded and *D*E-cad staining was used to follow the junctional domain in order to measure the apical surface area in selected cells (blue dots). Scale bar 10 μm (I) Quantification of the ratio of Crb accumulation in the different apical regions. n = 30 cells from 7 control embryos, n = 67 cells from 9 EGFR^DN^ embryos (J) Quantification of the area of the apical region. n = 69 cells from 6 control embryos, n = 60 cells from 5 EGFR^DN^ embryos.

In a first set of experiments we used the tracheal system. We had previously shown that in tracheal cells EGFR plays a role in regulating the subcellular accumulation of Crb in the apical domain (either in the SAR or in the AFR), and that the tracheal expression of a constitutively active form of EGFR, EGFR^CA^, leads to a loss of Crb in the SAR and a concomitant increased accumulation in the AFR (Fig 4A and 4B, S3D and S3E Fig) [20]. Quantification of the ratio of Crb accumulation in the SAR versus the AFR in control and EGFR^CA^ expressing tracheal cells clearly indicated a significant decrease in the mutant condition (Fig 4C) [20]. We now extended this analysis and investigated the effect of EGFR^CA^ at the cellular level measuring the apical surface area of DT cells. This analysis also indicated a clear difference with the control (Fig 4D), with cells expressing EGFR^CA^ showing a smaller apical area (Fig 4A'' and 4B'', S3E Fig). We previously documented higher levels of Crb in the trachea in EGFR^CA^ conditions when compared to wild type [20], pointing to a correlation between the subcellular accumulation of Crb in the SAR (rather than the amount of Crb) and apical membrane expansion. Interestingly, the loss of enrichment of Crb in the SAR was also accompanied by a loss of detectable anisotropic Crb accumulation, likely because preventing the normal Crb enrichment in the SAR (which depends on Crb intracellular trafficking [20]), prevents any possible subsequent anisotropic enrichment. Actually, quantification of Crb accumulation in LCJs and TCJs showed a ratio close to 1 (Fig 4E**),** indicating a rather uniform accumulation of Crb in EGFR^CA^ conditions. Furthermore, the analysis of the orientation of DT cells showed a shift towards the circumferential axis when compared to the wild type (Fig 4F**)**, indicating an abnormal cell expansion. The defects found in EGFR^CA^ conditions are similar in some aspects to those detected in Src42A loss of function conditions, and support the hypothesis that an anisotropic accumulation of Crb in the SAR promotes an anisotropic cell expansion.

In a second set of experiments we used another tubular epithelial tissue, the salivary gland (SG), to investigate whether Crb subcellular accumulation and apical expansion were also correlated. We found that in SGs of control embryos, and in contrast to the tracheal tissue, Crb was high in the AFR, with less distinct accumulation in the SAR (Fig 4G, S3F Fig). Because in the trachea EGFR plays a role in Crb subcellular accumulation, we explored if it also controlled Crb accumulation in the SG. Interestingly, when we blocked EGFR activity in the SG, by expressing EGFR^DN^, we found a clear relocalisation and accumulation of Crb in the SAR (Fig 4H and S3G). SG cells expressing EGFR^DN^ showed increased apical surface area (Fig 4H'' and 4J) which was accompanied by abnormal SG morphology (S3G Fig). The levels of Crb in EGFR^DN^ conditions were not increased when compared to control embryos (S3H Fig), suggesting again that it is the subcellular localisation of Crb in the apical domain, rather than the amount of Crb, that controls the apical area in the two tubular structures analysed.

Altogether these results confirm a role of EGFR in regulating the accumulation of Crb in the SAR or AFR, at least in tubular organs, as we already proposed [20]. We showed that EGFR regulates the trafficking of different cargoes, in particular Crb and Serp [20], raising the possibility that the regulation of the apical surface area depends on targets different than Crb. However, the fact that Serp is not present in the SGs and that Crb has already been proposed to promote apical membrane growth [32,33], strongly suggest that Crb is at least one of the targets downstream of EGFR regulating apical expansion. On the other hand, the results correlate apical cell expansion with Crb subcellular localisation in the SAR. We suggest that Crb accumulation in the SAR of LCJs could promote their expansion facilitating the elongation of the cell along the longitudinal axis, in agreement with the proposed role of Crb promoting apical membrane expansion [32,33]. Previous observations such as the expansion of the photoreceptor stalk membrane upon Crb overexpression [32] support this hypothesis, indicating that this can be a general mechanism.

### Crb promotes tube growth downstream of Src42A

**Fig 5:**
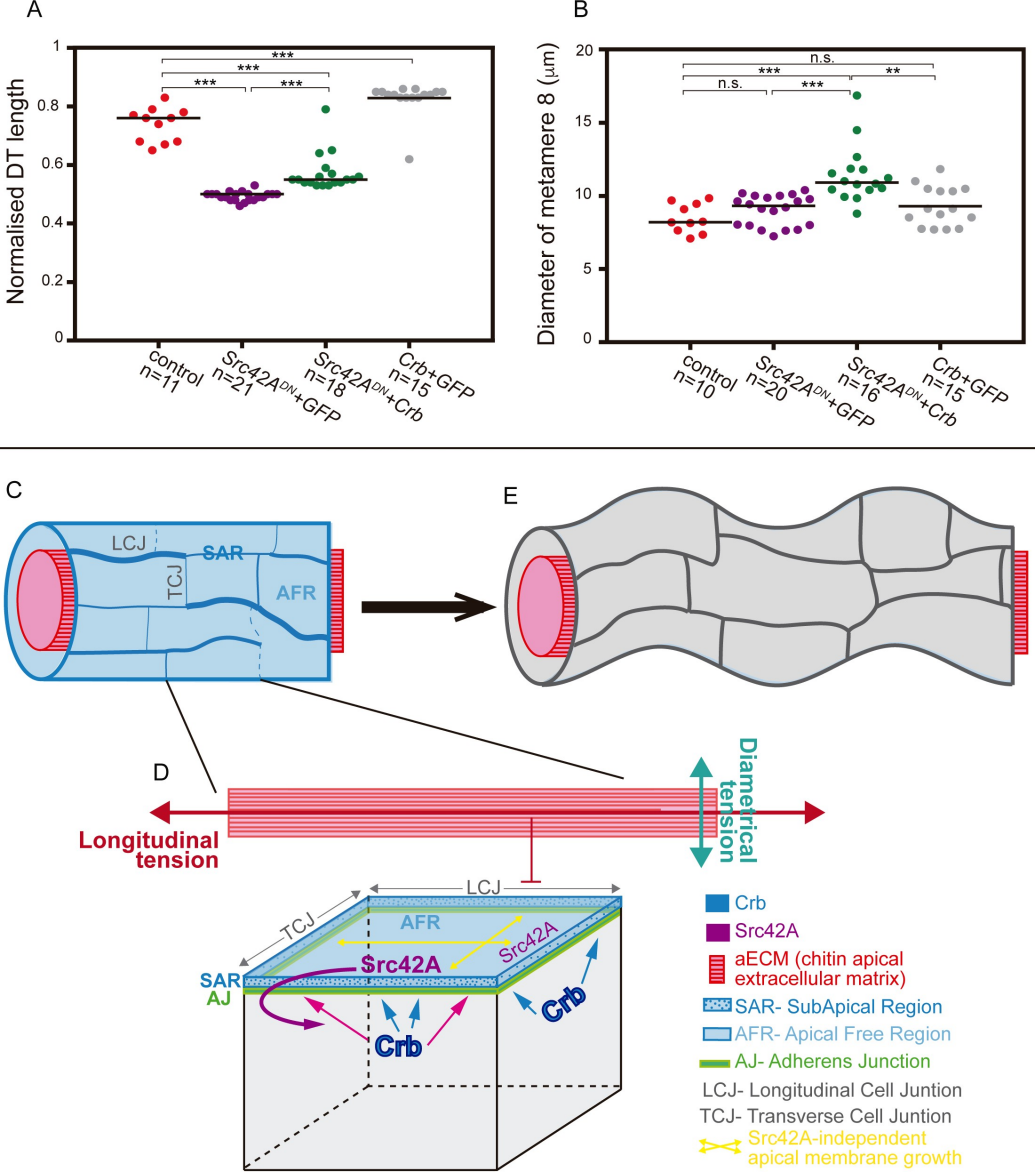
Genetic interaction between Crb and Src42A and model for DT elongation. (A) Quantification of DT length expressed as a ratio (length of the DT between transverse connectives 2–10 with respect embryo length) in control and different mutant conditions. The tracheal expression of Src42A^DN^ gives rise to 33% shrinkage of the DT, while a weak overexpression of Crb in the trachea produce a 12% enlargement of the tube. When Crb is overexpressed in Src42ADN mutant background, it promotes a significant 16% increase of tube length. (B) Quantification of the diameter of tracheal metamere 8 in control and mutant conditions. A mild increase of DT diameter is observed when Crb or Src42A^DN^ are expressed in tracheal cells. When both transgenes are combined, they produce a significant increase of tube diameter, indicating a circumferential growth of the tube. (C-E) Model: different mechanisms regulate the DT elongation. Crb protein accumulates apically in tracheal cells and becomes enriched in the SAR during tube elongation through a mechanism that is independent of Src42A (C and blue arrows in D). This base level of Crb may allow/promote an isotropic apical expansion of tracheal cells (yellow arrows in D). Secretion also promotes a Src42A independent mechanism of apical membrane growth (yellow arrows in D). The aECM restricts the growth of the tracheal tube along the longitudinal axis (red arrow in D). pSrc42A, accumulated at the apical region of the membrane, senses the intrinsic differential tension along the tube axes and/or the aECM (D). Src42A promotes an increased accumulation of Crb at LCJs (pink arrows), resulting in Crb anisotropy (C). Crb enrichment in the SAR of LCJs, in turn, promotes apical membrane expansion in the longitudinal direction, counteracting the restrictive activity of the aECM. The oriented cell elongation leads to the longitudinal tube growth (E).

Crb was proposed to regulate tube size by promoting apical membrane growth [15,16]. Accordingly, we found that a weak overexpression of Crb in an otherwise wild type background caused a mild increase in DT dimensions (a significant 12% enlargement of DT and a non-significant 9% diameter expansion) without perturbing the epithelial integrity and polarity (Fig 5A and 5B, S4A, S4B, S4G and S4H Fig). Src42A was shown to control tube elongation through interactions with dDaam and the remodelling of AJs [17,18]. We are now showing that Src42A regulates Crb levels, suggesting that Src42A may control tube elongation at least in part through regulation of Crb. Thus, we asked whether increased levels of Crb can bypass or compensate the requirement of Src42A in tube growth. To evaluate this possibility we performed genetic interaction experiments to test the ability of a weak Crb overexpression in a Src42A loss of function background. Interestingly, we found that Crb overexpression produced a partial but significant rescue of the short-DT phenotype of Src42A loss of function (Fig 5A, S4C and S4E Fig, Src42A^DN^ tracheal tubes elongate 16% when Crb is overexpressed). This result indicates that Crb acts downstream or in parallel of Src42A. Because, as we have described, we also observe that Src42A is required for Crb preferential enrichment at LCJs, we favour the hypothesis that Crb acts downstream of Src42A contributing to its function in tube elongation.

Remarkably, besides a rescue in DT length, we also detected an increase in the diameter of the tube when we overexpressed Crb in a Src42A loss of function background (a 25% expansion with respect to Src42A^DN^ mutants). Under these conditions, the DT diameter was not perfectly smooth and often showed dilations that were not detected in Src42A^DN^ or Crb overexpression conditions on their own. We interpret this isometric expansion of the DT along the diametrical and longitudinal directions as the result of an isotropic excess of Crb. Because in the absence of Src42A activity Crb accumulation is not properly polarised, this may promote a non-polarised increase of tube growth. To find support for this interpretation we analysed Crb accumulation in conditions of weak Crb overexpression. We detected high levels of Crb in the whole apical domain and in vesicles that precluded a proper analysis of Crb localisation and a systematic quantification of Crb accumulation. However, we could observe in examples in which we could detect a distinct accumulation of Crb that the overexpression of Crb in a wild type background leads to high enrichments of the protein particularly at LCJs (S4I and S4J Fig). This result suggests that the activity of Src42A biases the increased accumulation of Crb to the LCJs, correlating with a preferential growth mainly along the longitudinal axis (Fig 5A and 5B). In contrast, we detected a more generalised pattern of Crb overexpression in a Src42A loss of function mutant background (S4K and S4L Fig), consistent with the isometric tube growth observed (Fig 5A and 5B). In summary, although we could not directly test whether an anisotropic accumulation of Crb can exclusively compensate tube elongation in Src42A loss of function conditions (as we found no technical means to specifically localise Crb at the desired junctions), our results are consistent with the hypothesis that it is the anisotropic accumulation of Crb, regulated by Src42A, that mediates or promotes oriented tube growth along the longitudinal axis. Future experiments involving the generation of new tools designed to specifically localise Crb protein at desired subcellular domains will be needed to prove our model and to confirm an instructive and causal role of the here described anisotropic accumulation of Crb in cell elongation and polarised tracheal tube growth.

To summarise, here we find that Crb is transiently enriched in the SAR of DT cells in a polarised/anisotropic manner. This polarised distribution correlates with different dynamics or turnover of Crb protein, which appears to be more mobile and accumulate more at longitudinal junctions than at transverse ones. This polarised distribution also correlates with the anisotropic expansion of the apical membrane, axially-biased, that drives the longitudinal enlargement of the tracheal tubes. Interestingly we also find that Src42A is required for this anisotropic accumulation of Crb. Src42A was already known to regulate tube growth along the longitudinal axis, and we now propose that it performs this activity at least in part by promoting a Crb anisotropic enrichment. Src42A was also proposed to act as a mechanical sensor [17,18,30,34–36], translating the polarised cylindrical mechanical tension (an inherent property of cylindrical structures) into polarised cell behaviour [17]. Hence, we propose that Src42A would sense differential longitudinal/transverse tension stimuli and translate them into the cell by polarising Crb accumulation. It is likely that this Crb anisotropic accumulation in the SAR of LCJs mediates apical membrane expansion in the longitudinal direction, which would help to orient cell elongation and as a consequence longitudinal tube growth. A causal role for this Crb anisotropic accumulation in orienting cell elongation awaits definitive confirmation.

In light of our results and previously published work we propose the following model (Fig 5C–5E). Different mechanisms operate to regulate tube growth. On the one hand secretion drives apical membrane growth along the transverse axis independently of Src42A [17,37]. In addition, a basal level-pool of Crb accumulation independent of Src42A (this work) may promote or contribute to isotropic apical expansion [16]. On the other hand the presence of a properly organised luminal aECM also controls tube growth by restricting tube elongation [13,14,19]. A Src42A-dependent mechanism acts in coordination with these other mechanisms. Src42A would contribute to tube elongation through interactions with dDaam, the remodelling of AJs [17,18] and topping up Crb accumulation at LCJs with an enrichment-pool of Crb (this work). This increased accumulation of Crb at LCJs would bias the growth of the tube along the longitudinal axis, counteracting the restrictive activity of the aECM on tube elongation [13,14,19]. In the absence of Src42A activity, the Src42A independent mechanism/s of membrane growth would still operate, and would favour a compensatory growth along the transverse axis as observed [17,18], as diametrical growth is not restricted by the aECM.

The regulation of size and shape of tubular organs is important for organ function, as evidenced by the fact that loss of regulation can lead to pathological conditions such as polycystic kidney disease (PKD), cerebral cavernous malformation (CCM) or hereditary hemorrhagic telangiectasia (HHT) [8,38–40]. Src proteins have been implicated in malformations like PKD [41,42], highlighting the importance of investigating the mechanisms underlying their activities. While Src42A was proposed to regulate polarised cell shape changes during tracheal tube elongation through interactions with dDaam and the remodelling of AJs [17,18], no polarised downstream effectors have been identified up to date. Hence, identifying that Crb anisotropy is one of the downstream effects of Src42A activity adds an important piece to the puzzle. Src42A and Crb are conserved proteins with important roles in development and homeostasis and are involved in different pathologies [42,43]. This work provides an ideal model where to investigate the molecular mechanisms underlying their activities, their interactions, and their roles in morphogenesis.

## Materials and methods

### Fly stocks

The following stocks are described in Flybase: *y* ^*1*^*w* ^*118*^ (used as the wild type, WT, strain), *UAS-Egfr*^*DN*^, *UAS-Egfr*^*CA*^ (*UAS-Egfr*^*λtop*^) (kindly provided by M. Freeman), *Src42A*^*F80*^, *UAS-Src42A*^*DN*^, *UAS-Src42A* and *UAS-Src42A*^*CA*^ (kindly provided by S. Luschnig), and *UASCrbmini*-weak expression (kindly provided by E. Knust).

The *btlGal4* line (or *btlGal4-UAS-Src-GFP* to also mark the tracheal cells) was used to drive transgene expression in all tracheal cells from invagination onwards and *fkhGal4* to drive expression in the salivary glands. Blue or green balancers were used to identify the embryos of interest.

The knock in allele *Crb*^*GFP*^*-C* was kindly provided by Y. Hong.

Control or embryos expressing the transgenes were collected at 22–25°C or 29°C to control levels of overexpression.

### Immunofluorescent stainings

Immunostainings were performed on embryos collected on agar plates fixed for 20 minutes (except for *D*E-cad staining, for which embryos were fixed for 10 minutes) in 4% formaldehyde in PBS. The following primary antibodies were used: mouse anti-Crb (Cq4) (1:20) and rat anti-*D*E-cad (DCAD2) (1:100) from Developmental Studies Hybridoma Bank DSHB; rabbit anti-pSrc pY418 (1:50) from ThermoFisher; rabbit anti-GFP (1:600) from Molecular Probes and goat anti-GFP (1:600) from Roche, chicken anti-β-gal (1:200) from Abcam; rbb anti-Src42A (1:200) generously provided by T. Kojima; and rabbit anti-Serp (1:300) generously provided by S. Luschnig. Alexa Fluor 488, 555, 647 (Invitrogen) or Cy2-, Cy3-, Cy5- Conjugated secondary antibodies (Jackson ImmunoResearch) were used at 1:300 in PBT 0.5% BSA. CBP (Chitin Binding Protein) was visualised as a secondary antibody at 1:300.

### Image acquisition and morphometric analyses

Images shown are from embryos at stage 16 unless otherwise indicated. Fluorescence confocal images of fixed embryos were obtained with Leica TCS-SPE system using a 20x or a 63x (1.40–0.60 oil) objective. Unless otherwise indicated, images shown are projections of Z stacks sections (0.21–0.5 μm).

To measure DT dimensions, confocal projections of stage 16 embryos stained with CBP and Crb or *D*E-cad were used. To calculate DT length we traced a path following the DT using the freehand selection tool in Fiji between the junction DT/transverse connective from transverse connective 2 to 10. We measured the total length of the embryo and expressed DT length as a ratio DT length (from metamere 2 to 10)/embryo length. The diameter of metamere 8 was calculated as the average of three measurements along the metamere (anterior to the dorsal branch, and just next to the fusion points inside the metamere).

Images were imported into Fiji and Photoshop for measurements and adjustments, and assembled into figures using Illustrator.

### Analysis of protein accumulation in the junctions

Images of DT fragments between metameres 7 to 9 of stage 16 embryos were taken to analyse the Crb and *D*E-cad accumulation at cell junctions. After setting the longitudinal tube axis to 0°, cellular junctions were identified as longitudinal (LCJs, those oriented 0°±30° with respect to the axis) or transverse junctions (TCJs, oriented 90°±30°). Between 80–90% of junctions could be unequivocally assigned as LCJs or TCJs in all conditions analysed. The projection of the *D*E-cad channel was used to properly follow the cellular junctions to measure *D*E-cad and Crb protein accumulation. Accumulation of Crb or *D*E-cad was measured in all those junctions that were identified as LCJs or TCJs to avoid biased selection (i.e. in 80–90% of junctions in each metamere).

Because immunostaining experiments are not ideal to analyse protein levels in a quantitative manner, and in order to compare embryos from different independent immunostaining experiments, we expressed the accumulation of Crb and *D*E-cad as the ratio of accumulation at LCJs compared to TCJs for each embryo. To this end we generated a projection from the different stacks using the Max Intensity tool in the Fiji software. To measure the total fluorescence at each cell junction we obtained the "raw intensity density" (the sum of all fluorescence intensity of the selected junction) manually drawing the junctions using a 5-pixel line that included the whole junctional area. The fluorescence intensity of Crb or *D*E-cad of all LCJs or TCJs of each embryo was normalised to the total length of each type of junction. For each control or mutant embryo the ratio of fluorescence intensity/length at LCJ and TCJ was calculated for Crb and *D*E-cad. The ratios were compared between control and mutant conditions using the Scatter Plot tool of GraphPad Prism.

To analyse the total levels of Crb protein in the tracheal junctions to compare control and *Src42A* mutants we performed different independent experiments in which control (*Src42*^*F80*^/*CyO* heterozygotes) and mutant (*Src42*^*F80*^ mutants) embryos were collected, fixed and stained together. Confocal images were acquired with the same laser settings for each individual experiment. We generated a projection from the different stacks using the Max Intensity tool in the Fiji software and subtracted background. To measure the total Crb fluorescence at each cell junction we obtained the "raw intensity density" (the sum of all fluorescence intensity of the selected junction) manually drawing the junctions with a 5-pixel line that included the whole junctional area (using the *D*E-cad channel projection to properly follow the cellular junctions). The fluorescence intensity of Crb in LCJs or TCJs of each embryo was normalised to the total length of each type of junction. The fluorescence intensity/length were compared between control and *Src42A*^*F80*^ mutants conditions using the Scatter Plot tool of GraphPad Prism.

### Time lapse movies

Embryos at stage 15 (12–13 hours) were mounted and imaged for 1.30–2 hours, covering part of stage 16 (which lasts about 3 hours). Dechorionated embryos were mounted and lined up on a Menzel-Gläser cover slips with oil 10-S Voltalef (VWR) and covered with a membrane (YSI membrane kit). Life imaging was performed on a Zeiss Lsm780 Confocal and Multiphoton System. A 950 nm Multiphoton laser MaiTai HP DS was used to image embryos using an oil 63x/1.4 NA objective. To visualize time-lapse movies of typically two tracheal metameres, maximal intensity projections were generated in Fiji software.

### FRAP assay

*btlGal4*; *Crb*^*GFP*^*-C* (i.e. *control*) and *btlGal4-UASSrc42A*^*DN*^; *Crb*^*GFP*^*-C* (i.e. mutant) embryos were collected at 29°C. Embryos were mounted and lined up on Menzel-Gläser cover slips with oil 10-S Voltalef (VWR) and covered with a Teflon membrane (YSI membrane kit). FRAP was carried out on a Zeiss Lsm780 Confocal and Multiphoton System. The ZEN 2.1 SP3 of the Zeiss Confocal Software was used for data acquisition. The 488 nm emission line of an Argon laser was used for excitation at 1–2% power. We selected embryos at the appropriate stage (stage 15–16) oriented in a lateral position. We performed 10 pre-bleach scans with the 63x objective with a 1.5 zoom. These pre-bleach scans covered several Z-sections (1μm thick, 4 sections) in order to image the Crb accumulation at cell junctions that lie at different focal planes. Regions-of-interest (ROIs) of the same size were selected at longitudinal and transverse junctions. After 10 pre-bleach scans, bleaching was performed at these ROIs at high laser power (100 iterations at 100% power). Post-bleach scans were obtained immediately after bleaching at every 10 seconds and during 10–20 minutes. Average projections of the Z-sections were exported for each time point and assembled into a movie using Fiji software. The StackReg plugin in ImageJ was used to correct the movement of the embryo in the xy plane.

Fluorescence intensity in the ROIs was measured at each time point with Fiji software intensity plot profile tool. Igor Pro software was used for normalization, curve fitting (single-exponential fit), and calculation of recovery half-time (t_1/2_) and the mobile fraction (M_f_).

The FRAP experiments were technically challenging. Although we performed FRAP for many (>20) ROIs from various embryos for each genotype, the trachea often moved during imaging, so many bleached ROIs were lost during recording. In addition, to FRAP ROIs at specific junctions in tracheal cells (with lengths typically ranging from 2,5 to 9 μm) was often difficult. Only movies where the ROIs remained in focus throughout (assessed by the fact that neighbouring junctions remained in focus) are reported here. We performed one single FRAP experiment per embryo. Kymographs were generated using the KymographBuilder plugin of the Fiji software, after image denoising (Gaussian blurring).

### Crb subcellular accumulation

We quantified the total levels of Crb (using the Sum Fluorescence Intensity projection in Fiji) in different apical subcellular domains of embryos at stage 16. We selected individual cells from a region in the DT between metameres 7 and 9 or in the SG and generated projections of a few sections to include only the whole cell or a small number of them. We quantified Crb accumulation in the SAR by outlining the cell contour (using *D*E-Cad to visualise it) drawing a 6-pixel line on the junctional area and measuring the signal within each line. To measure Crb in AFR, a section inside the cell (defined by *D*E-cad junctional outline) was drawn with the freehand tool of Fiji. We expressed the subcellular accumulation as the ratio between SAR/AFR.

To analyse the total levels of Crb protein in salivary glands we added the values of the SAR and the AFR for each individual cell. We compared control (*btlsrcGFP*) and mutant (*fkhGal4-UASEGFR*^*DN*^) embryos that were collected, fixed and stained together. Confocal images were acquired with the same laser settings for each individual experiment. The fluorescence intensity was compared between control and mutants conditions using the Scatter Plot tool of GraphPad Prism.

### Apical surface area measurements

We analysed the apical surface area of stage 16 tracheal and salivary gland cells, typically the same ones for which we analysed Crb accumulation in the SAR and the AFR. We obtained projections from several stacks covering the whole tube using the Max Intensity tool in Fiji. We used *D*E-cad staining to outline the whole apical surface. To analyse the apical surface area avoiding inaccurate measurements due to deformations caused by tube curvature, we used a semi-automated ImageJ macro developed at IRB ADM facility by Sebastien Tosi. The algorithm unwounded the thin tubes to a 2D plane. Assuming that the sample can be modelled as a 3D bended tube of constant diameter, the image was straighten first along the medial axis of the sample before computing the radial projection. Since the sample can be both bended within the XY plane (acquisition view) and perpendicularly to it, we performed the straightening in two steps: first in the original 3D stack (XY view) and then in the XZ view of the resulting stack. For both steps we manually drew the medial axis as a polyline used to resample the 3D stack following the tube shape (IJ Straighten). Next, we drew a ring capturing the layer of interest in a 3D stack oriented along the straightened medial axis of the sample. Finally, the radial projection was computed along this ring for each slice of the 3D stack (IJ getProfile). In the projected image, the vertical position develops along the medial (longitudinal) axis of the sample while the horizontal position develops along the ring (circumferential axis). Importantly, to ensure no aspect ratio distortion, all 3D stacks were resampled to isotropic voxel size as a first step. The projected images were replicated horizontally to allow the measurement of cells intersecting a vertical image edge. We quantified the apical surface area by tracing a polygon following *D*E-cad staining in the unfolded images. We expressed the apical surface area in μm^2^.

### Cell orientation

To analyse the orientation of DT cells we measured the angle between the longest axis of the cell (corresponding to the longest axis after fitting ellipses to the cells) and the direction of the A-P axis of the tube. The measurements were computed automatically using Image J from the 2-D images of unfolded tubes, where the vertical position (90°) develops along the longitudinal axis. To represent cell orientation we grouped the measured orientation of the cells into 6 intervals (I-VI): cells in interval I were oriented from 0 to 30 degrees; in interval II from 30°-60°; in interval III from 60°-90°; in interval IV from 90°-120°; in interval V from 120°-150° and in interval VI from 150°-180°. Cells oriented 180°-360° degrees are undistinguishable from those oriented in 0°-180°, and are therefore already included in one of the six intervals. A radial chart (Excel) was used to represent the frequency of each interval; each interval was located at one of the vertices of the chart.

### Quantifications and statistics

Total number of cells/embryos is provided in text and figures. Error bars indicate standard error (s.e.) or standard deviation (s.d) as indicated. *p*-values were obtained with an unpaired two-tailed Student’s *t*-test using STATA 12.1 software. *p<0.05, 0.001>**p<0.01, ***P<0.001.

## Supporting information

S1 Fig Analysis of Src42A loss of function. (A-C) Further examples of Crb and *D*E-cad accumulation in control and loss of function conditions. Images show lateral views of stage 16 embryos of indicated genotypes stained for the indicated markers. The colour-coded fluorescence intensity shown for *D*E-cad and Crb matches the heat map shown on the right. Note that in contrast to the control (A), the fluorescence intensity in LCJs or TCJs for *D*E-cad and also for Crb is more homogeneous in *Src42A* mutants (B) or when Src42A is downregulated (C). Scale bar 10 μm
(D,E) Lateral views of stage 16 wild type embryos stained for Src42A protein or activated pSrc42A and Crb. Images show single confocal sections in the case of Src42A stainings (D,D';F,F'. The general pattern of Src42A prevents to obtain a defined image in projections of sections), or projections of several confocal sections covering the apical domain of the DT. Z-reconstructions (z in D,E, corresponding to single confocal sections) show co-localisation of Crb and Src42A or pSrc42A in the SAR (marked by ellipses). Thin cross lines indicate the position of the z-reconstruction. Src42A protein accumulates in cell membranes (D') while pSrc42A localises at the apical membrane region (E'). Scale bar D 10 μm, E 7,5 μm
(F,G) Lateral views of stage 16 *Src42A* mutant embryos. Src42A protein is still localised at the membrane in mutants (F,F' single confocal section), while the activated pSrc42A signal is lost (G,G', projection of confocal sections). Scale bar F 10 μm, G 7,5 μm
(H) Lateral view of a stage 16 embryo stained for activated pSrc42A and Crb. Image shows a projection of confocal sections. Note the presence of activated pSrc42A homogeneously in the apical domain and the absence of polarised distribution (H'').
Scale bar 5 μm.
(TIF)

S2 Fig Analysis of *Crb^GFP^* FRAP experiments. (A) Comparison of the Mobile fraction observed at LCJs and TCJs in control embryos and in embryos in which Src42A is downregulated in the trachea. The Mf at control LCJs is significantly higher than in other conditions. Note that when Src42A is downregulated the Mf at LCJ decreases to levels similar to those of TCJs, suggesting that Src42A is required to increase the Mf precisely at the LCJs.
(B) Comparison of the Half-time (t_1/2_). The results indicate a similar kinetics of recovery in all conditions analysed.
(C) Comparison of the average fit of normalised post-bleach recovery of fluorescence from the different FRAP experiments at LCJs and TCJs in control and Src42A downregulation conditions. Note the higher recovery at LCJs of control embryos and the similar recovery at LCJs and TCJs in mutants and at control TCJs.
(D,F,H,J) Normalised curves of all FRAP experiments showing the levels of fluorescence relative to the pre-bleach levels during the cycles of image acquisition (a cycle every 10 sec). The samples were photobleached at cycle 10. The curves for each experiment (embryo) are shown in colours (red for control LCJs, orange for control TCJs, green for Src42^DN^ LCJs and bue for Src42^DN^ TCJs) and the average of the FRAP curves for each experimental condition is shown in black.
(E,G,I,K) Images acquired before (time 0 min) and after (from time 10 min) photobleaching a region in LCJs or TCJs (red arrows) are shown. The initial fluorescence intensity is shown in heat maps at the left. Kymographs of the bleached areas are shown. Asterisks mark the bleached region. The X axis represents time and the Y axis represents distance.
Error bars indicate standard error (s.e.). Scale bar 10 seconds.
n = 7 LCJs and n = 6 TCJs from wild type embryos, and n = 7 LCJs and n = 9 TCJs from mutant embryos.
(TIF)

S3 Fig Protein accumulation in trachea and salivary glands. (A) Lateral view of stage 16 embryos expressing Src42A in tracheal cells. Note the faint accumulation of Crb in the SAR, preferentially at LCJs. Scale bar 10 μm
(B,C) Lateral views of stage 15 embryos. Images show single confocal sections. Control (*btlGal4-UASGFP*, B) and embryos overexpressing Src42A (*btlGal4-UASGFP+UASSrc42A*, C) are labelled with GFP to visualise the tracheal cells and with Mega, a Septate Junction marker. Note that while in control embryos Mega accumulates at the basolateral membrane, when Src42A is overexpressed it expands to apical and basal regions (red arrows in C'). Scale bar 10 μm
(D,E) Lateral views showing a region of the DT of stage 16 embryos of indicated genotypes stained for the indicated markers. When EGFR is constitutively activated Crb accumulation in the SAR is less conspicuous and cells do not expand as in the control. Images show projections of several confocal sections. Scale bar 7,5 μm
(F,G) Lateral views showing a region of the salivary gland of stage 16 embryos of indicated genotypes stained for the indicated markers. Crb is localised in the whole apical area in control embryos (F') but when EGFR is downregulated it gets enriched in the SAR (G'). Images show projections of several confocal sections. Scale bar 5 μm
(H) Scatter Plot comparing the total levels of Crb protein in SG cells (accumulation in the SAR+AFR) in control embryos and in embryos expressing EGFR^DN^. Measurements were performed in two different independent experiments in which mutant and control embryos were processed together. Crb levels in the mutant condition are similar or lower to those in the control. In experiment 1, n = 16 SG cells from 2 wild type embryos and n = 43 cells from 5 mutant embryos. In experiment 2, n = 13 cells from 2 wild type embryos and n = 13 cells from 2 mutant embryos.
(TIF)

S4 Fig Genetic interactions between Src42A and Crb. Lateral views of stage 16 embryos of indicated genotypes stained for Crb (green or white) and CBP (red) to visualise the tracheal lumen.
(A,C,E,G) DT length was measured tracing a line following DT shape from transverse connective (TC) 2 to 10. DT length was normalised to embryo length. Note the short DT in loss of function conditions for Src42A (K) and the elongation in mild overexpression conditions for Crb (O). Crb can partially restore DT elongation in Src42A loss of function conditions (M). Scale bar 7,5 μm
(B,D,F,H) DT diameter was measured in the tracheal metamere 8. Note the expansion of the DT diameter when Crb and Src42A^DN^ are both expressed in tracheal cells (N). Scale bar 10 μm
(I-L) Weak Crb overexpression leads to increased and generalised accumulation of the protein in the whole apical domain and in vesicles. This pattern prevents the analysis of Crb accumulation in most cases. However, in examples in which we can detect a distinct accumulation of Crb, we observed high and clear enrichments of Crb protein at LCJs in a wild type background (red arrows in I,J). In contrast, in a Src42A loss of function background Crb accumulation was also high in TCJs (blue arrows in K,L).
(TIF)

S1 Movie Crb accumulation during tube elongation. Embryo carrying *Crb^GFP^* visualised from a lateral view using an inverted Zeiss Lsm780 confocal with 63x Oil objective and a 2 zoom. Images were taken every 3 minutes during 1,30 hours in 20 Z-stack of 0,5μm, from late stage 15 to mid stage 16. Note the enrichments of Crb-GFP protein preferentially at LCJs.
(AVI)

S2 Movie FRAP experiment in control LCJ. Region of a DT of a stage 16 *btlGal4; Crb^GFP^-C* embryo visualised from a lateral view using an inverted Zeiss Lsm780 confocal with 63x Oil objective and a 1,5 zoom. After several pre-bleach scans, a LCJ (marked with white arrow) was bleached. Post-bleach scans were taken immediately every 10 seconds for 10 minutes.
(AVI)

S3 Movie FRAP experiment in control TCJ. Region of a DT of a stage 16 *btlGal4; Crb^GFP^-C* embryo visualised from a lateral view using an inverted Zeiss Lsm780 confocal with 63x Oil objective and a 1,5 zoom. After several pre-bleach scans, a TCJ (marked with white arrow) was bleached. Post-bleach scans were taken immediately every 10 seconds for 14 minutes.
(AVI)

S4 Movie FRAP experiment in a LCJ in Src42A mutant embryos. Region of a DT of a stage 16 *btlGal4-UASSrc42A^DN^; Crb^GFP^-C* embryo visualised from a lateral view using an inverted Zeiss Lsm780 confocal with 63x Oil objective and a 1,5 zoom. After several pre-bleach scans, a LCJ (marked with white arrow) was bleached. Post-bleach scans were taken immediately every 10 seconds for 10 minutes.
(AVI)

S5 Movie FRAP experiment in a TCJ in Src42A mutant embryos. Region of a DT of a stage 16 *btlGal4-UASSrc42A^DN^; Crb^GFP^-C* embryo visualised from a lateral view using an inverted Zeiss Lsm780 confocal with 63x Oil objective and a 1,5 zoom. After several pre-bleach scans, a TCJ (marked with white arrow) was bleached. Post-bleach scans were taken immediately every 10 seconds for 12 minutes.
(AVI)
