# Supplementary figures and images for: Anisotropic Crb accumulation, modulated by Src42A, is coupled to polarised epithelial tube growth in Drosophila
Source: PLoS Genet. 2018 Nov 26;14(11):e1007824. doi: 10.1371/journal.pgen.1007824 (PMC6283610; doi:10.1371/journal.pgen.1007824)

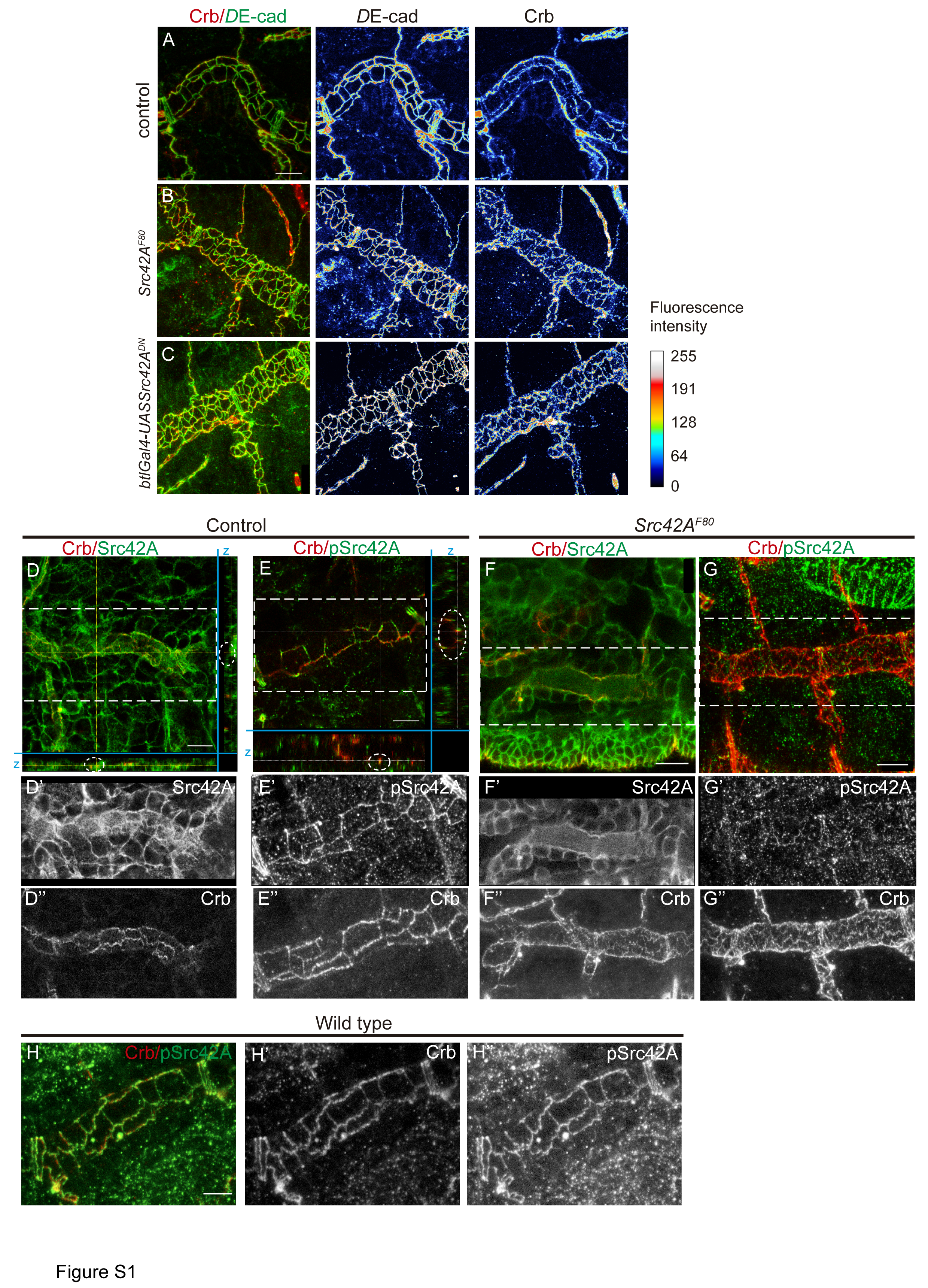

Supplement: S1 Fig — (A-C) Further examples of Crb and DE-cad accumulation in control and loss of function conditions. Images show lateral views of stage 16 embryos of indicated genotypes stained for the indicated markers. The colour-coded fluorescence intensity shown for DE-cad and Crb matches the heat map shown on the right. Note that in contrast to the control (A), the fluorescence intensity in LCJs or TCJs for DE-cad and also for Crb is more homogeneous in Src42A mutants (B) or when Src42A is downregulated (C). Scale bar 10 μm (D,E) Lateral views of stage 16 wild type embryos stained for Src42A protein or activated pSrc42A and Crb. Images show single confocal sections in the case of Src42A stainings (D,D';F,F'. The general pattern of Src42A prevents to obtain a defined image in projections of sections), or projections of several confocal sections covering the apical domain of the DT. Z-reconstructions (z in D,E, corresponding to single confocal sections) show co-localisation of Crb and Src42A or pSrc42A in the SAR (marked by ellipses). Thin cross lines indicate the position of the z-reconstruction. Src42A protein accumulates in cell membranes (D') while pSrc42A localises at the apical membrane region (E'). Scale bar D 10 μm, E 7,5 μm (F,G) Lateral views of stage 16 Src42A mutant embryos. Src42A protein is still localised at the membrane in mutants (F,F' single confocal section), while the activated pSrc42A signal is lost (G,G', projection of confocal sections). Scale bar F 10 μm, G 7,5 μm (H) Lateral view of a stage 16 embryo stained for activated pSrc42A and Crb. Image shows a projection of confocal sections. Note the presence of activated pSrc42A homogeneously in the apical domain and the absence of polarised distribution (H''). Scale bar 5 μm. (TIF) [file pgen.1007824.s001.tif]

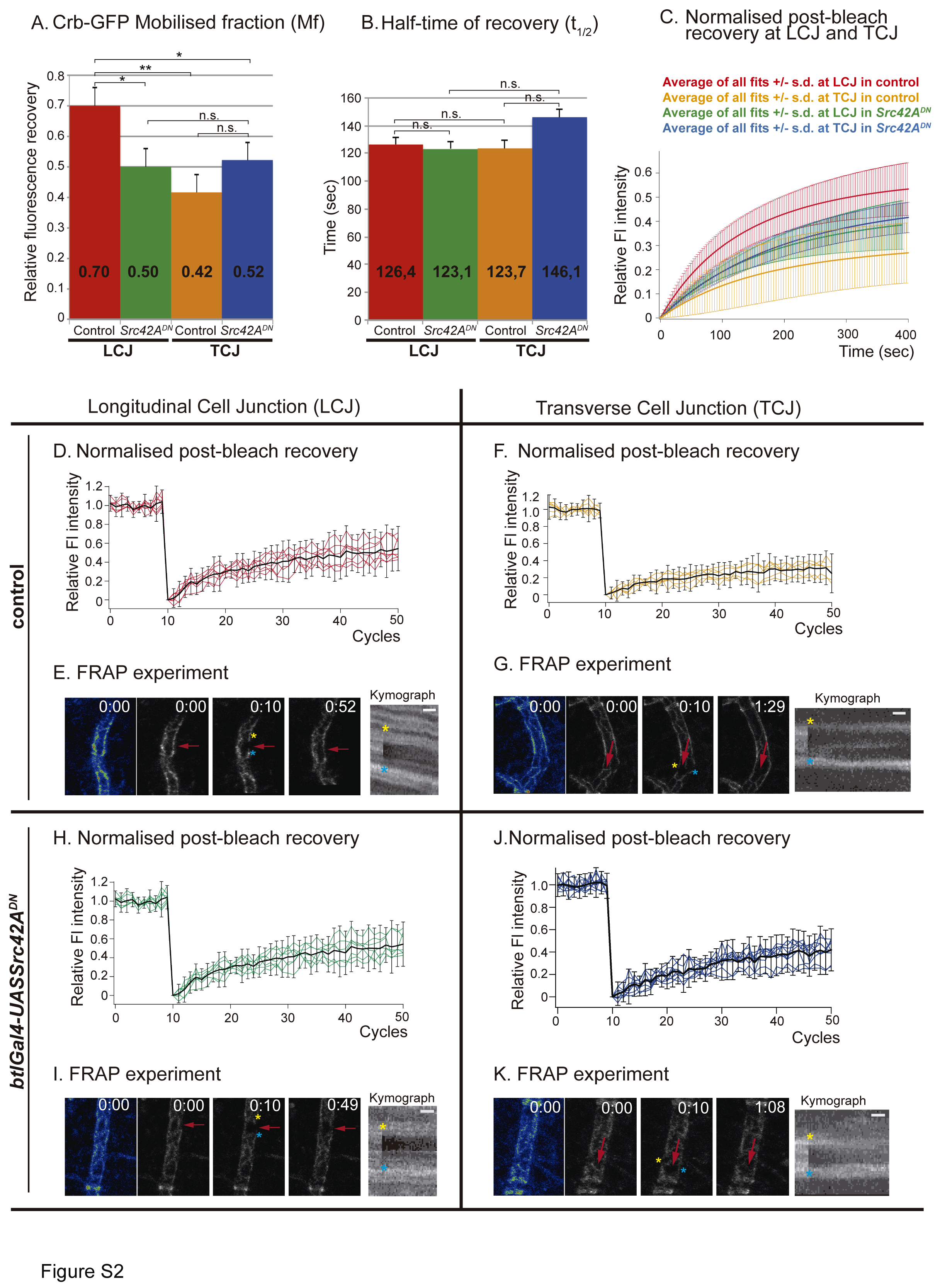

Supplement: S2 Fig — (A) Comparison of the Mobile fraction observed at LCJs and TCJs in control embryos and in embryos in which Src42A is downregulated in the trachea. The Mf at control LCJs is significantly higher than in other conditions. Note that when Src42A is downregulated the Mf at LCJ decreases to levels similar to those of TCJs, suggesting that Src42A is required to increase the Mf precisely at the LCJs. (B) Comparison of the Half-time (t1/2). The results indicate a similar kinetics of recovery in all conditions analysed. (C) Comparison of the average fit of normalised post-bleach recovery of fluorescence from the different FRAP experiments at LCJs and TCJs in control and Src42A downregulation conditions. Note the higher recovery at LCJs of control embryos and the similar recovery at LCJs and TCJs in mutants and at control TCJs. (D,F,H,J) Normalised curves of all FRAP experiments showing the levels of fluorescence relative to the pre-bleach levels during the cycles of image acquisition (a cycle every 10 sec). The samples were photobleached at cycle 10. The curves for each experiment (embryo) are shown in colours (red for control LCJs, orange for control TCJs, green for Src42DN LCJs and bue for Src42DN TCJs) and the average of the FRAP curves for each experimental condition is shown in black. (E,G,I,K) Images acquired before (time 0 min) and after (from time 10 min) photobleaching a region in LCJs or TCJs (red arrows) are shown. The initial fluorescence intensity is shown in heat maps at the left. Kymographs of the bleached areas are shown. Asterisks mark the bleached region. The X axis represents time and the Y axis represents distance. Error bars indicate standard error (s.e.). Scale bar 10 seconds. n = 7 LCJs and n = 6 TCJs from wild type embryos, and n = 7 LCJs and n = 9 TCJs from mutant embryos. (TIF) [file pgen.1007824.s002.tif]

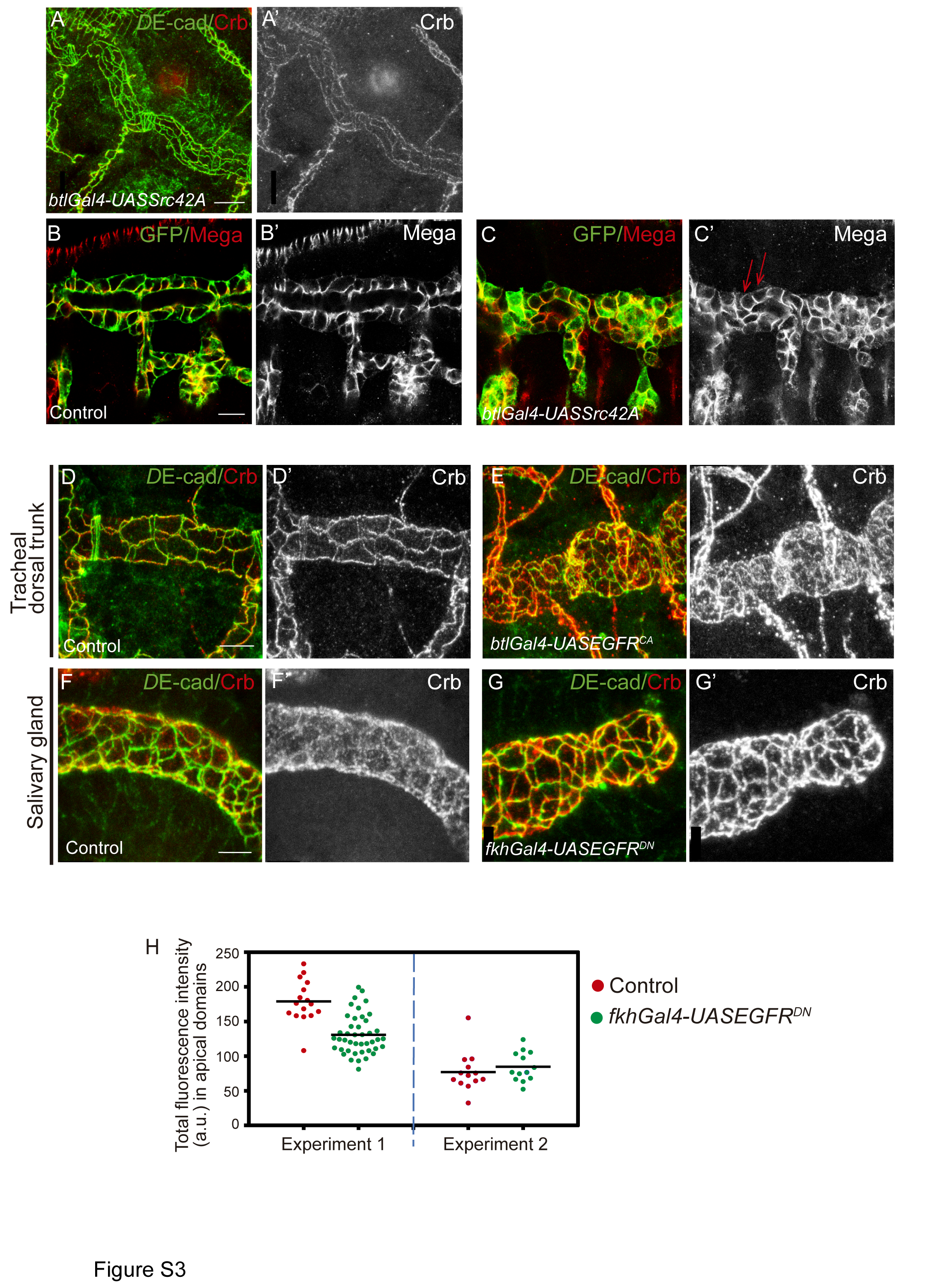

Supplement: S3 Fig — (A) Lateral view of stage 16 embryos expressing Src42A in tracheal cells. Note the faint accumulation of Crb in the SAR, preferentially at LCJs. Scale bar 10 μm (B,C) Lateral views of stage 15 embryos. Images show single confocal sections. Control (btlGal4-UASGFP, B) and embryos overexpressing Src42A (btlGal4-UASGFP+UASSrc42A, C) are labelled with GFP to visualise the tracheal cells and with Mega, a Septate Junction marker. Note that while in control embryos Mega accumulates at the basolateral membrane, when Src42A is overexpressed it expands to apical and basal regions (red arrows in C'). Scale bar 10 μm (D,E) Lateral views showing a region of the DT of stage 16 embryos of indicated genotypes stained for the indicated markers. When EGFR is constitutively activated Crb accumulation in the SAR is less conspicuous and cells do not expand as in the control. Images show projections of several confocal sections. Scale bar 7,5 μm (F,G) Lateral views showing a region of the salivary gland of stage 16 embryos of indicated genotypes stained for the indicated markers. Crb is localised in the whole apical area in control embryos (F') but when EGFR is downregulated it gets enriched in the SAR (G'). Images show projections of several confocal sections. Scale bar 5 μm (H) Scatter Plot comparing the total levels of Crb protein in SG cells (accumulation in the SAR+AFR) in control embryos and in embryos expressing EGFRDN. Measurements were performed in two different independent experiments in which mutant and control embryos were processed together. Crb levels in the mutant condition are similar or lower to those in the control. In experiment 1, n = 16 SG cells from 2 wild type embryos and n = 43 cells from 5 mutant embryos. In experiment 2, n = 13 cells from 2 wild type embryos and n = 13 cells from 2 mutant embryos. (TIF) [file pgen.1007824.s003.tif]

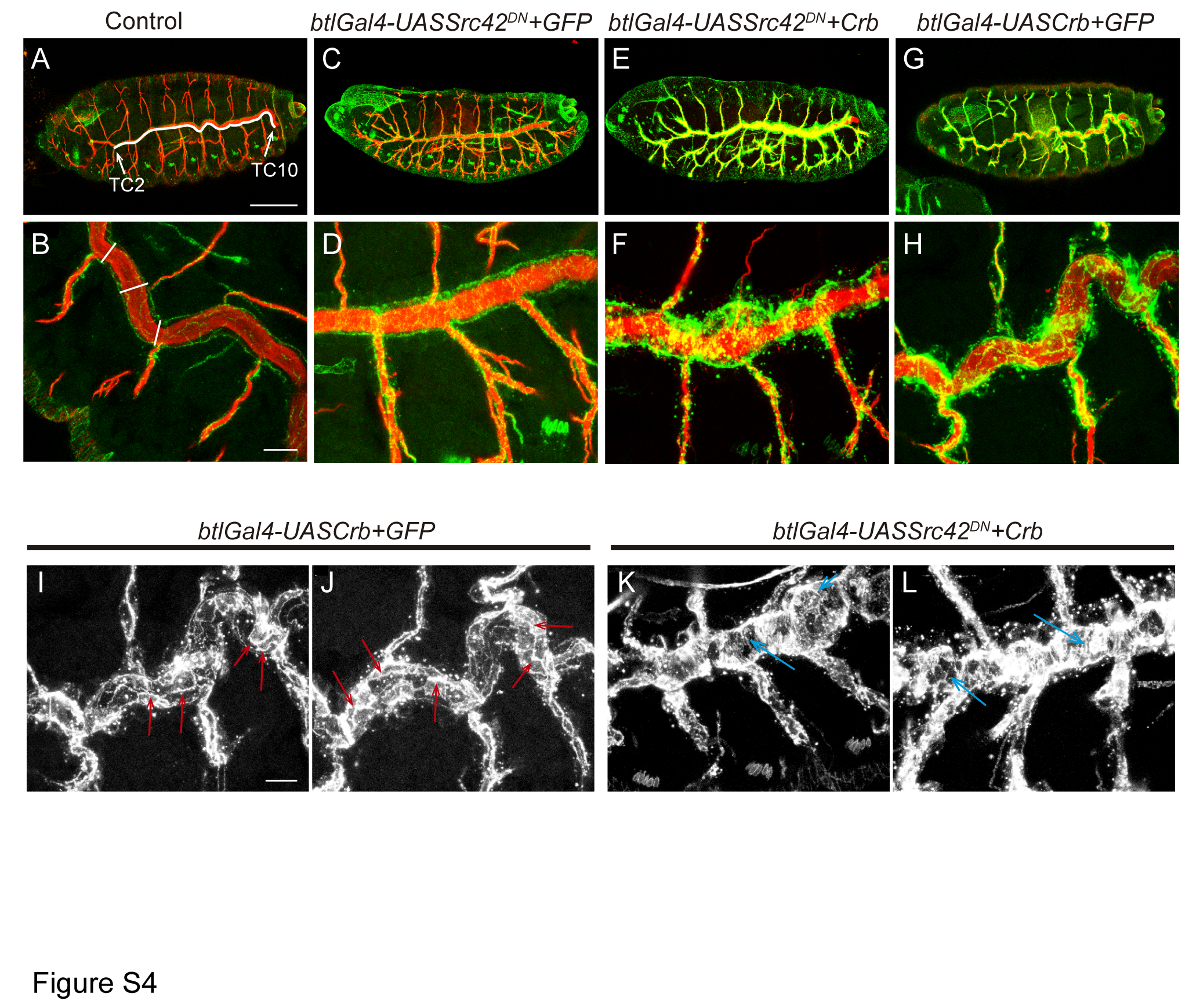

Supplement: S4 Fig — Lateral views of stage 16 embryos of indicated genotypes stained for Crb (green or white) and CBP (red) to visualise the tracheal lumen. (A,C,E,G) DT length was measured tracing a line following DT shape from transverse connective (TC) 2 to 10. DT length was normalised to embryo length. Note the short DT in loss of function conditions for Src42A (K) and the elongation in mild overexpression conditions for Crb (O). Crb can partially restore DT elongation in Src42A loss of function conditions (M). Scale bar 7,5 μm (B,D,F,H) DT diameter was measured in the tracheal metamere 8. Note the expansion of the DT diameter when Crb and Src42ADN are both expressed in tracheal cells (N). Scale bar 10 μm (I-L) Weak Crb overexpression leads to increased and generalised accumulation of the protein in the whole apical domain and in vesicles. This pattern prevents the analysis of Crb accumulation in most cases. However, in examples in which we can detect a distinct accumulation of Crb, we observed high and clear enrichments of Crb protein at LCJs in a wild type background (red arrows in I,J). In contrast, in a Src42A loss of function background Crb accumulation was also high in TCJs (blue arrows in K,L). (TIF) [file pgen.1007824.s004.tif]
